# Supplementary figures and images for: Comparing the impact of vaccination strategies on the spread of COVID-19, including a novel household-targeted vaccination strategy
Source: PLoS One. 2022 Feb 2;17(2):e0263155. doi: 10.1371/journal.pone.0263155 (PMC8809548; doi:10.1371/journal.pone.0263155)

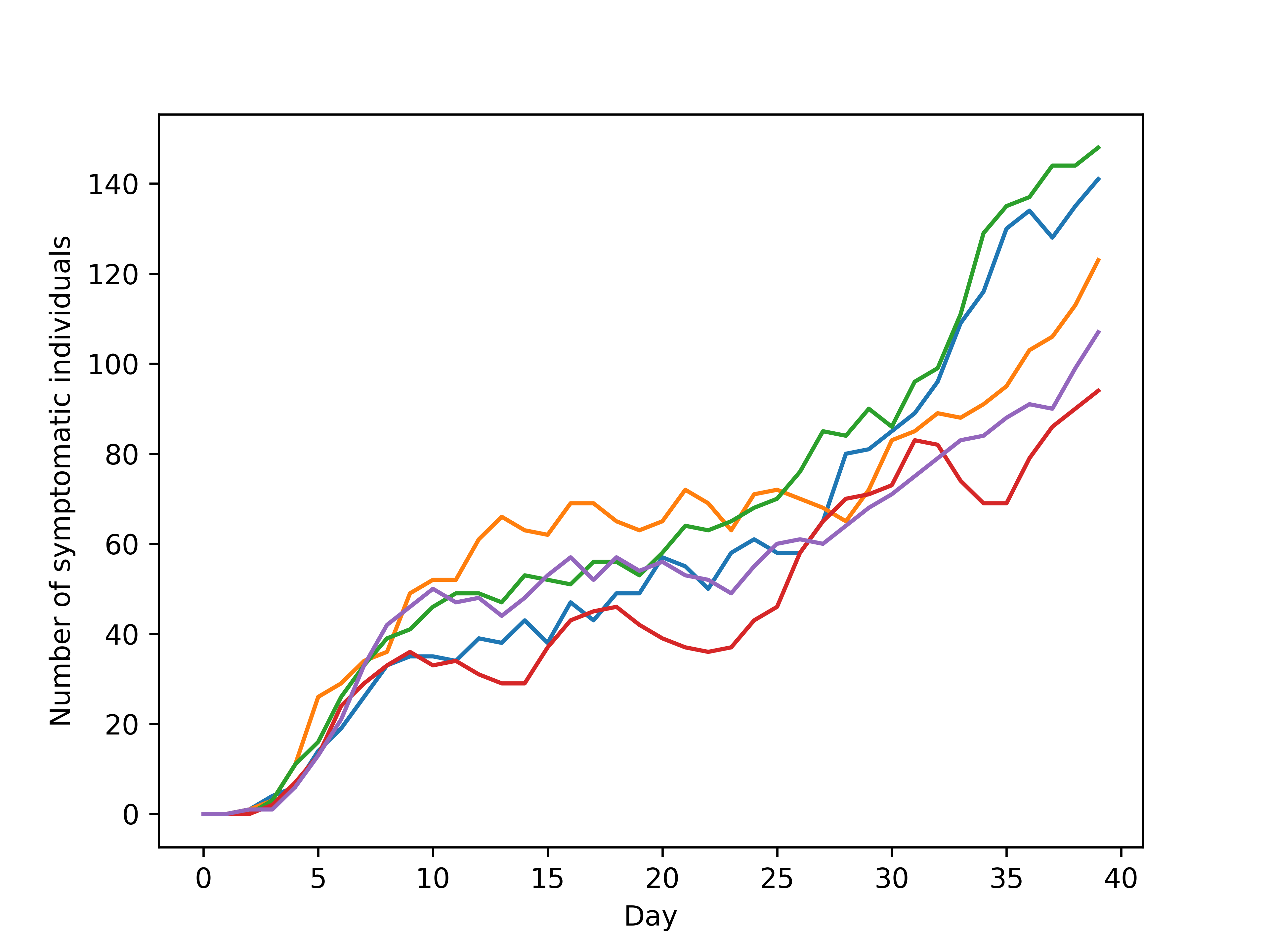

Supplement: S1 Fig — Each run corresponds to a similar initialization with different random seeds. While individual curves evolve stochastically, each replicate is characterised by an steep rise from around 3-4 days into the simulation (corresponding to the incubation time), which slows down to a more moderate exponential trend from about 10 days from the start of the simulation. (PNG) [file pone.0263155.s001.png]

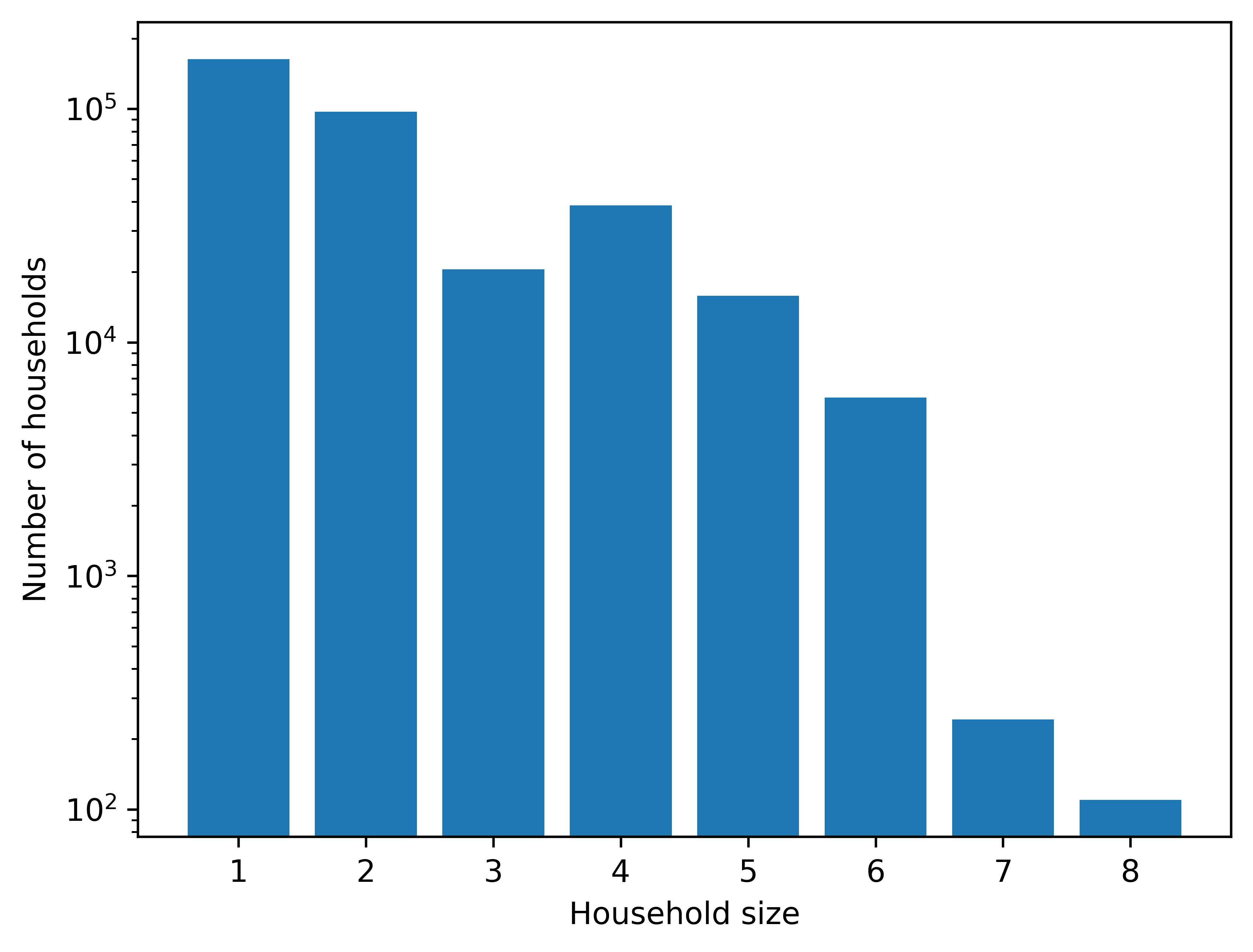

Supplement: S2 Fig — Bars represent the amount of households in the computational model with the number of members indicated on the x-axis. (PNG) [file pone.0263155.s002.png]

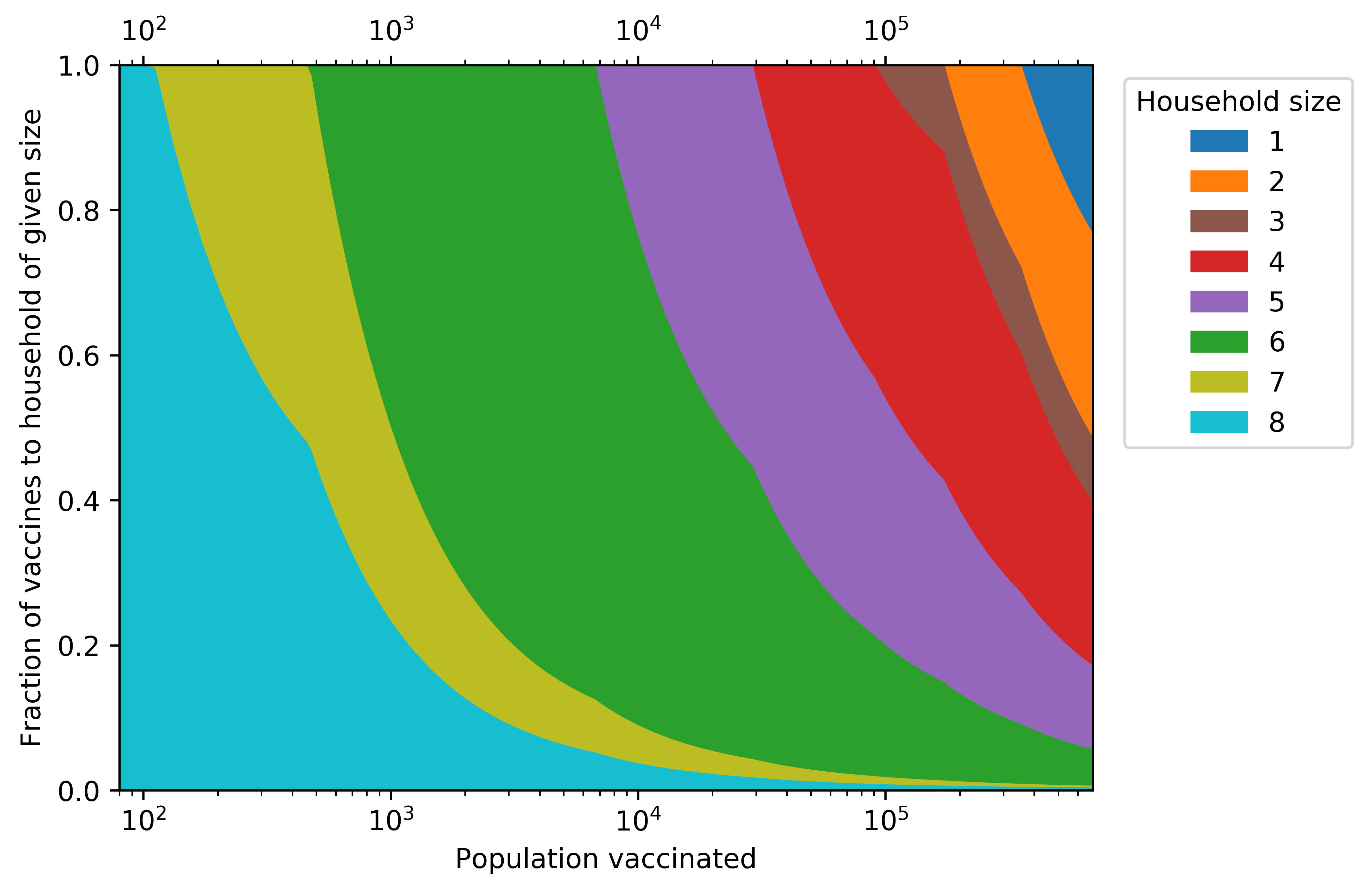

Supplement: S3 Fig — Illustrating the EHR strategy on our Oslo model, we begin by allocating one vaccine to each of the ≈100 households of size 8. This means that all of the first ≈100 individuals vaccinated are members of 8-person households. The second stage in deployment consists of allocating the next ≈350 doses evenly (one each) to each of the ≈100 households of size 8 and ≈250 households of size 7, until a total of ≈450 individuals are vaccinated. In the third stage (individuals ≈450 through ≈6700), one more vaccine is allocated to each household of size 6 (≈5800 households), 7 or 8. This is repeated until all individuals are vaccinated, with more and more households included in each stage (by order of decreasing size). While members of large households make up the entirety of the first people vaccinated, their relative share of the vaccinated population drops as vaccine deployment progresses. (PNG) [file pone.0263155.s003.png]

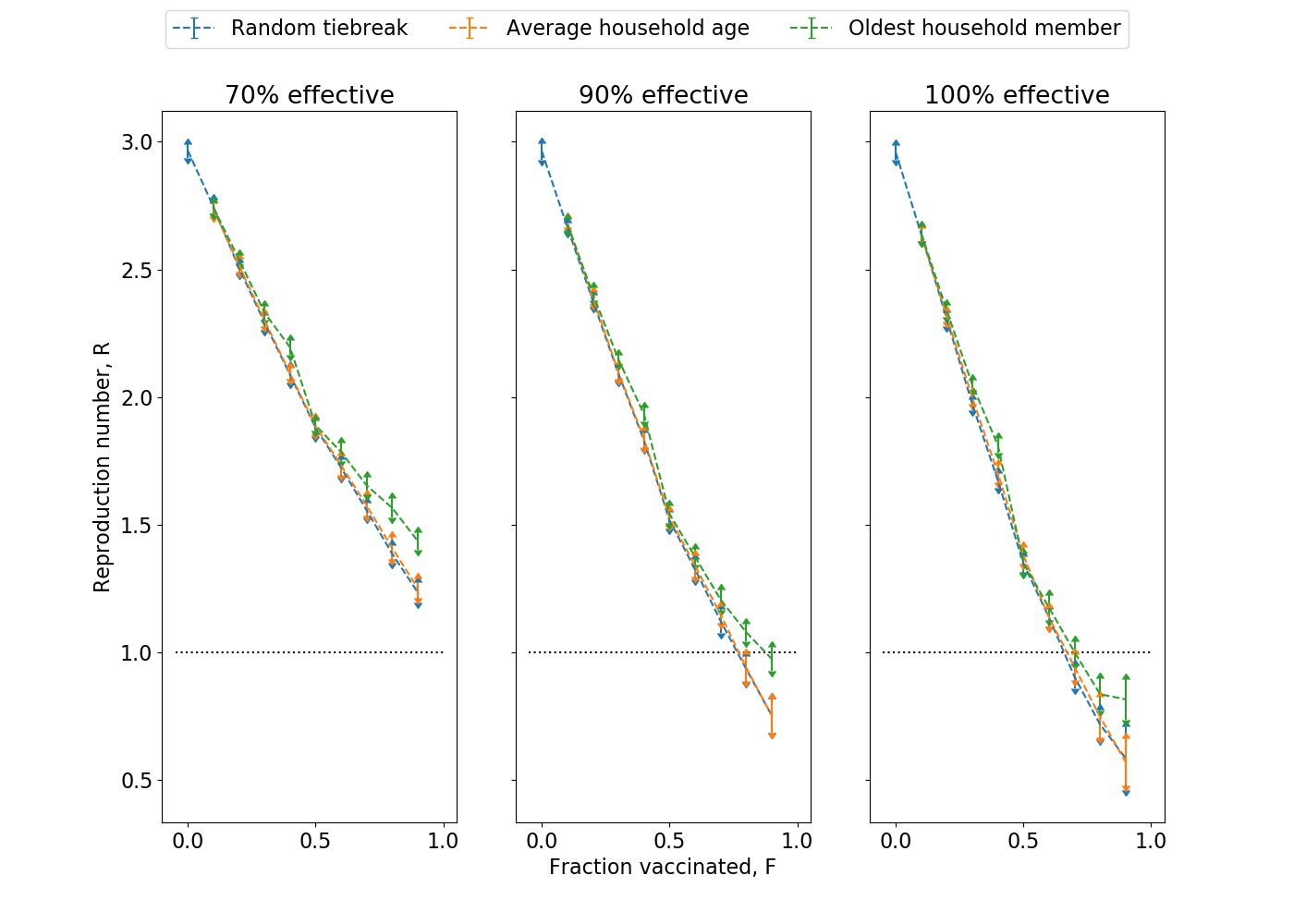

Supplement: S4 Fig — Computationally determined reproduction numbers according to vaccinated fraction of population and choice of tie-breaking strategy for same-sized households in the EHR strategy, assuming the Delta variant with a pre-vaccine reproduction number of R0 = 3. Random tiebreak and average household age are approximately equivalent, while priorizing households with older members performs slightly worse. (PNG) [file pone.0263155.s004.png]

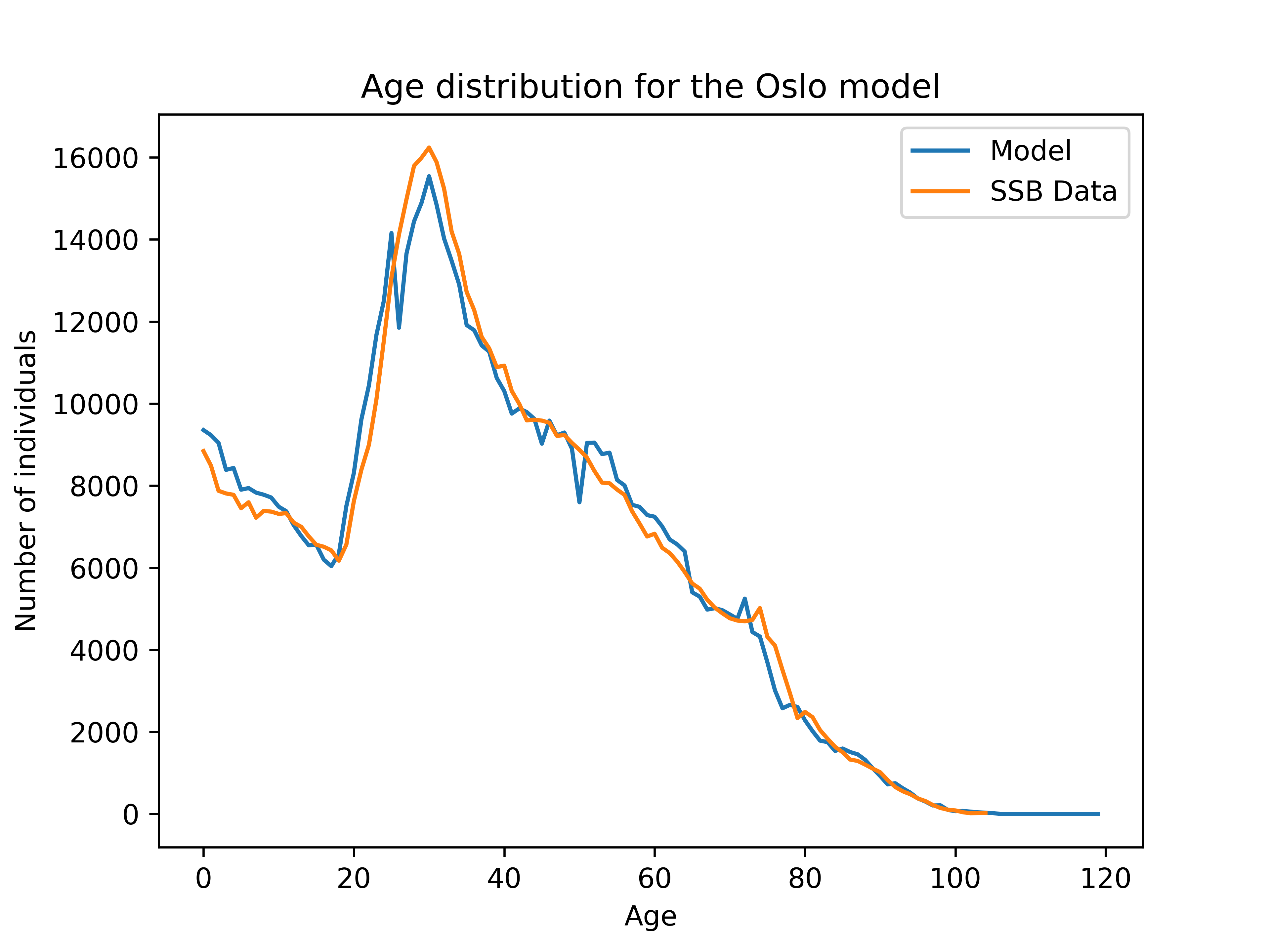

Supplement: S5 Fig — Number of Oslo inhabitants of a given age in the procedurally generated model, compared with real-life data provided by Statistics Norway (SSB), illustrating a generally close correlation. (PNG) [file pone.0263155.s005.png]

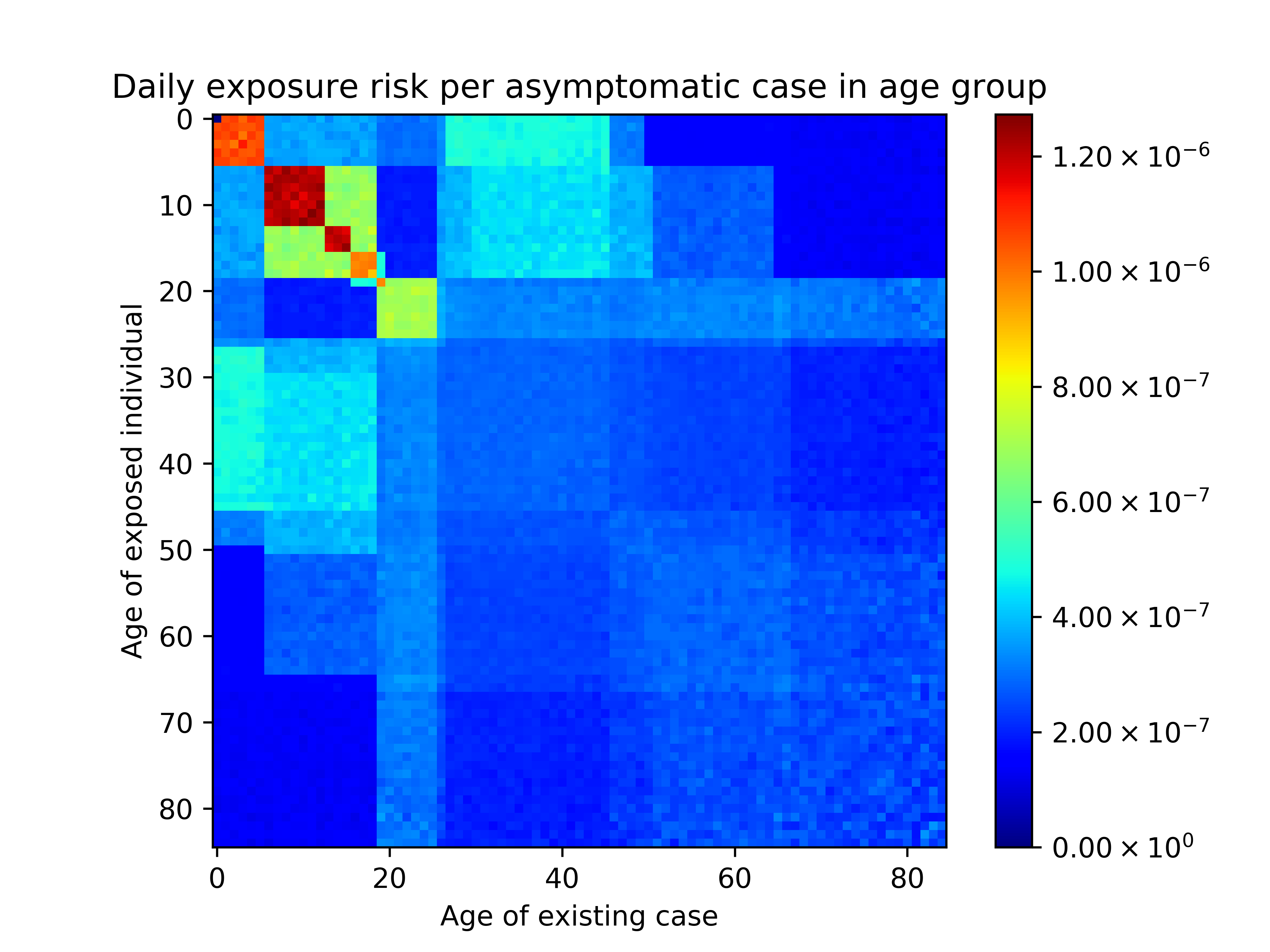

Supplement: S6 Fig — Each pixel indicates average the daily infection probability for each individual of age X resulting from one asymptomatic individual of age Y (assuming a regime with the Alpha variant at R = 3.2). This probability consists of the sum across all layers of shared clique memberships between individuals of age X and age Y multiplied with the daily probability of infection for the corresponding layer, divided by the the product of the number of individuals of ages X and Y, resulting in a symmetrical matrix. While similar, this is not strictly a contact matrix as in [23–26], as these contact matrices do not differentiate between the probability of transmission for different types of contact. (PNG) [file pone.0263155.s006.png]

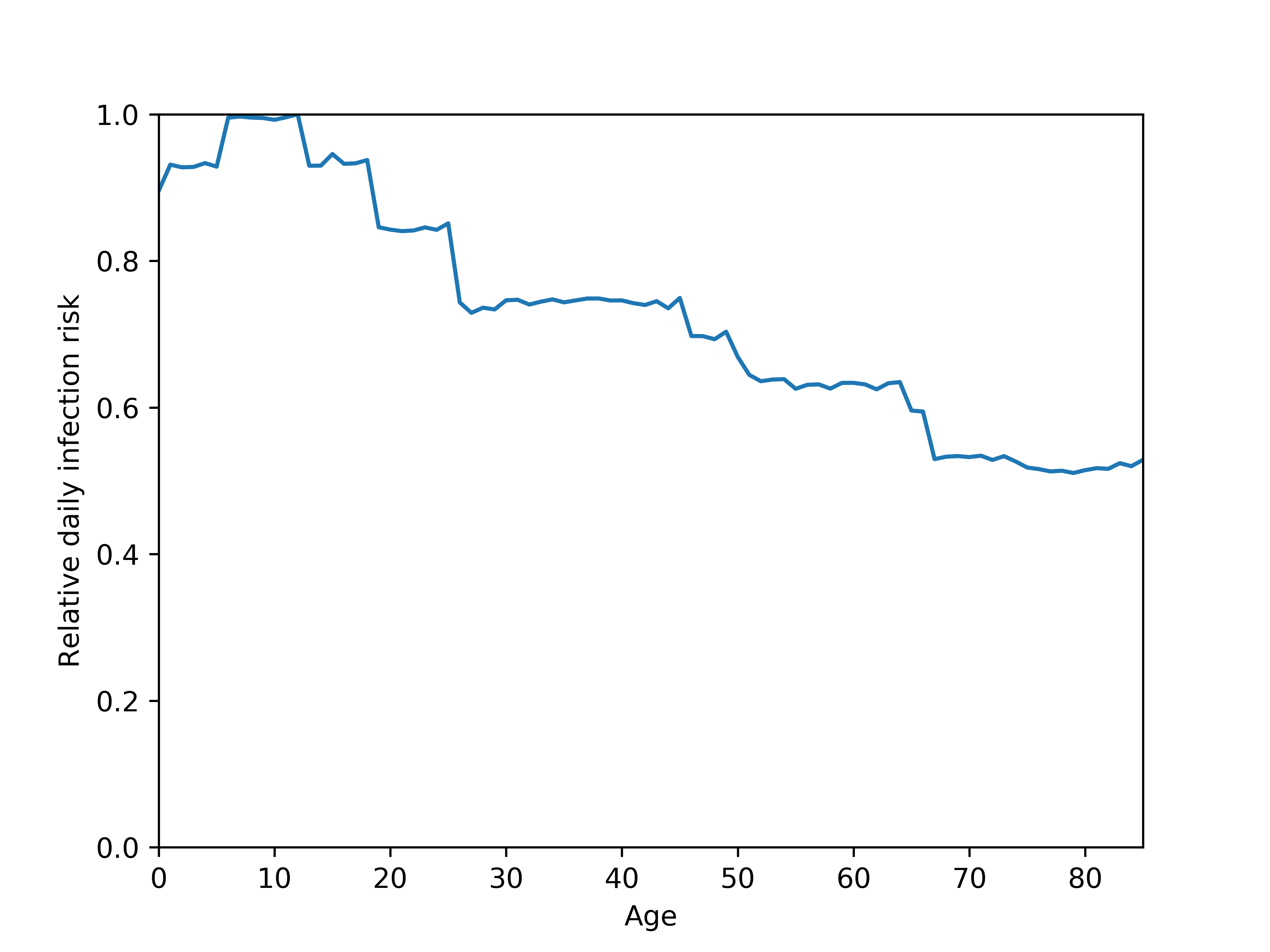

Supplement: S7 Fig — Relative risk is defined as the average expected number of daily infections caused by an asymptomatic individual (assuming a regime with the Alpha variant at R = 3.2) in an entirely susceptible population, divided by the corresponding value for an asymptomatic 12-year old (0.29). Due to the symmetrical nature of contacts, this can also be interpreted as the relative daily risk of a susceptible individual of a given age becoming infected, assuming a uniform proportion of infectious individuals for all ages. (PNG) [file pone.0263155.s007.png]
