# Supplementary material for: Comparing the impact of vaccination strategies on the spread of COVID-19, including a novel household-targeted vaccination strategy
Source: PLoS One. 2022 Feb 2;17(2):e0263155. doi: 10.1371/journal.pone.0263155 (PMC8809548; doi:10.1371/journal.pone.0263155)
Supplement: Attachment — Submitted filename: review_PlosOne_sep2021_Norway_network_model.pdf [file pone.0263155.s008.pdf]

The authors use a recently published multi-layer network model to evaluate the effectiveness of selected COVID-19 vaccination strategies. Using their model, they can show that a strategy that targets large households (minimizing the maximal number of unvaccinated individuals in any household) is better than a random strategy, which in turn is better than age-focused strategies. These differences seem particularly strong for high vaccine effectiveness and low basic reproductive numbers.
While the overall premise of the work is interesting and the manuscript is well-written, I have substantial issues, mainly with the methodology and the description of the model (lack of important information, only available in supplement of original published paper), both of which should be addressed prior to acceptance.
Major comments / concerns:

- The findings heavily depend on model assumptions and parameter choices of the original, already published model (mainly in the supplement of the published paper). I read through the published paper and supplement to better understand the model and findings. The authors should briefly describe the key parameters that influence/bias their findings in the methods and/or discussion (limitation) section. Without that information, this manuscript is a nice read with interesting results but cannot be blindly trusted as there are numerous model assumptions that may give rise to the specific findings, in particular why EHR is so much better than age-based vaccine allocation. Just some possibly important pieces of information include:
    1. the distribution of household sizes in Oslo (a graph that displays vaccine coverage versus effective household size could also be helpful)
    2. details regarding the social contact network: is the POLYMOD data (Mossong et al., 2008; Prem et al., 2017 - ref 25) used to inform the age-age-contact structure? If so, how does this translate to the distribution of the number of cliques people of a certain age are part of? I believe the authors did not use the POLYMOD data. In that case, I wonder: How does the age-assortativity that the authors obtain intrinsically in their social clique network compare to the inferred age-assortatitivity for Norway (Prem et al., 2017 - ref 25).
    3. the parameter used for the average time in the exposed, pre-symptomatic and symptomatic compartments. The authors only write "the number of cases will briefly dip after about one generation (9 days) as most of the initial cases recover within a few days from each other". If an exponential distribution is used for the transition from compartment to compartment, this may be generally true, however the time from infection to recovery varies (sum of two or three exponential random variables) and the statement "as most of the initial cases recover within 94 a few days from each other" is very vague/inaccurate.
    4. The authors write "each individual also comes into contact with a random group of individuals chosen from the entirety of the municipal population". How large is this random group? Is it the same size for all individuals? Is there heterogeneity, or at least age-based heterogeneity?
- The authors use a neat approach to model the social interaction network: cliques representing schools, companies, etc., (same contacts every day) as well as daily changing random contacts. Schools and companies are of vastly different size and the authors use available data from Oslo to inform the distribution of these entities. However, modeling large entities (schools or companies) as complete graphs where each individual has a daily contact with each other leads to issues that may strongly influence the results. The authors tuned their model so that the number of infections stemming from households, schools, work, etc. match Norwegian public health data (page 11 of supplement of ref 8). However, this matching means that individuals at small entities hardly contribute to the epidemic and that the epidemic is largely driven by those individuals that are part of a large clique. While this seems intuitive in general, the assumption of a complete graph and the same infection rates (Supp Table 1 in ref 8), irrespective of size of the clique, troubles me. The authors acknowledge this in page 5 of suppl of ref 8, "other layers, such as schools and workplaces, are not likely to actually see this form of all-to-all contact on any given day. This is reflected in a substantially lower combined contact and infection probability for these layers". Small schools and workplaces likely see all-to-all contacts on a given day, while large schools and workplaces do not. Since the number of contacts (edges) in a k-clique increases quadratically in k, the authors' assumption of same infection probabilities for a certain entity seems wrong and leads to a model where too much of the epidemic spread happens at large entities.

- The findings, as they stand, would be huge news. However, the manuscript lacks a sensitivity analysis that investigates how the differences shift when the society is in a strict lockdown vs normal operation. In Figures 2 and 3, the authors vary R0, the basic reproductive number prior to vaccination (called unvaccinated R), but only by varying the random contact level. If this variation of R0 is to represent the different states of social distancing, school, work, etc contacts must also be varied. In particular, how do the results change if the society opens (as it happened in the United States) and proportionately fewer of the contacts happen at the household level? The authors only investigate a fixed reduction in contacts at each entity and it seems that the values used are no longer accurate: from their previous paper (ref 8, suppl, page 11), "all schools and day care facilities close, as do 50% of workplaces. Random contacts are reduced by 83%". Schools are no longer closed. I would be very interested in seeing a 2-D sensitivity analysis where the contact level at schools/workplaces as well as random contacts are varied.

Minor comments / concerns:

- Why is the effective household size reduced by exactly one and not by the respective vaccine effectiveness, wouldn't that be a more appropriate measure?
- How much better would the following modified EHR strategies perform compared to EHR? Would the improvement (over random assignment of the last doses) only be marginally better?
    1. assign last doses (lines 152-155) to those households (one each) with the oldest unvaccinated individuals
    2. assign last doses (lines 152-155) to those households (one each) with the highest average age
- How many times was the stochastic model run for each parameter setting? This needs to be mentioned.
