# Supplementary material for: Comparing the impact of vaccination strategies on the spread of COVID-19, including a novel household-targeted vaccination strategy
Source: PLoS One. 2022 Feb 2;17(2):e0263155. doi: 10.1371/journal.pone.0263155 (PMC8809548; doi:10.1371/journal.pone.0263155)
Supplement: Attachment — Submitted filename: Response to Reviewers.pdf [file pone.0263155.s009.pdf]

Response to Reviewers

General Comments

We thank the Reviewers for their positive assessments of our work, and the Editor for the chance to submit a revised version to PLoS One.

Review Comments to the Author

Reviewer #1:

The authors use a recently published multi-layer network model to evaluate the effectiveness of selected COVID-19 vaccination strategies. Using their model, they can show that a strategy that targets large households (minimizing the maximal number of unvaccinated individuals in any household) is better than a random strategy, which in turn is better than age-focused strategies. These differences seem particularly strong for high vaccine effectiveness and low basic reproductive numbers.

While the overall premise of the work is interesting and the manuscript is well-written, I have substantial issues, mainly with the methodology and the description of the model (lack of important information, only available in supplement of original published paper), both of which should be addressed prior to acceptance.

We thank the Reviewer for their interest in our manuscript, positive comments and the thorough effort made in reviewing.

Major comments / concerns:

The findings heavily depend on model assumptions and parameter choices of the original, already published model (mainly in the supplement of the published paper). I read through the published paper and supplement to better understand the model and findings. The authors should briefly describe the key parameters that influence/bias their findings in the methods and/or discussion (limitation) section.

Without that information, this manuscript is a nice read with interesting results but cannot be blindly trusted as there are numerous model assumptions that may give rise to the specific findings, in particular why EHR is so much better than age-based vaccine allocation. Just some possibly important pieces of information include:

In light of the Reviewer's comments, we agree with the Reviewer that a more thorough description of the model would be useful to readers. We have therefore added a set of supplementary figures, while still aiming to keep the figures presented in the main manuscript focused on the results of our analysis.

1. the distribution of household sizes in Oslo (a graph that displays vaccine coverage versus effective household size could also be helpful)

In order to provide a more thorough description of Oslo's household structure, and how this relates to the EHR strategy, we included two relevant figures in the supplementary materials. S2 Fig describes the distribution of household sizes in Oslo according to our model, as well as official statistics compiled by Statistics Norway (SSB). As a variation on the second requested figure, S3 Fig illustrates the fraction of

vaccines allocated to households of different sizes as a function of the number of vaccinated individuals. We hope that the Reviewer also finds this figure helpful and illustrative.

2. details regarding the social contact network: is the POLYMOD data (Mossong et al., 2008; Prem et al., 2017 - ref 25) used to inform the age-age-contact structure? If so, how does this translate to the distribution of the number of cliques people of a certain age are part of? I believe the authors did not use the POLYMOD data. In that case, I wonder: How does the age-assortativity that the authors obtain intrinsically in their social clique network compare to the inferred age-assortativity for Norway (Prem et al., 2017 - ref 25).

The Reviewer is correct that POLYMOD data was not used to inform the age-age contact structure. Age assortativity in the model is simply a consequence of clique composition data combined with assumptions made concerning how different age groups participate in different layers, some of which are quite precise (such as ages of schoolchildren), while other assumptions must be generally broad (such as age distributions within workplaces). In a similar vein, the random contact layer is not assortative between individuals of similar ages (although an individual's activity in the random layer is somewhat age-dependent). While it could theoretically be possible to use POLYMOD data to increase the fidelity of the random layer, this would result in a prohibitive increase in its computational requirements given the current implementation.

We agree with the Reviewer that age-dependent contact patterns in our model are highly relevant to understanding the effect of different strategies, and have therefore included several figures (S5-S7 Fig) and relevant discussion (lines 325-339) to describe this in our supplementary text. We observe that while many qualitative aspects of age-assortativity are generally consistent, our patterns also differ somewhat from the POLYMOD data in terms of resolution and in exact quantitative ratios. It is, however, important to keep in mind that the weighting of contacts according to frequency and duration such as reported by POLYMOD does not necessarily directly correspond to transmission risks for disease in general and COVID-19 in particular. We therefore believe that our calibration approach -relying on demographic data combined with COVID-19 transmission statistics according to type of contact- remains a sound option, even if the resulting contact matrix ends up rather coarse.

3. the parameter used for the average time in the exposed, pre-symptomatic and symptomatic compartments. The authors only write "the number of cases will briefly dip after about one generation (9 days) as most of the initial cases recover within a few days from each other". If an exponential distribution is used for the transition from compartment to compartment, this may be generally true, however the time from infection to recovery varies (sum of two or three exponential random variables) and the statement "as most of the initial cases recover within 94 a few days from each other" is very vague/inaccurate.

The Reviewer is correct in that since recovery time is a sum of multiple random variables, there is substantial spread in disease durations. In order to elaborate on this, we have included S1 Fig, showing how the epidemic curve progresses immediately after case seeding. We still see a noticeable dip or flattening out in cases about one generation in, corresponding to an approximately simultaneous recovery of the initial seed cases.

4. The authors write "each individual also comes into contact with a random group of individuals chosen from the entirety of the municipal population". How large is this random group? Is it the same size for all individuals? Is there heterogeneity, or at least age-based heterogeneity?

The Reviewer raises an important question. We have now expanded our description of the model's random layer (lines 62-71), and thank the Reviewer for directing us towards this improvement to the manuscript.

The authors use a neat approach to model the social interaction network: cliques representing schools, companies, etc., (same contacts every day) as well as daily changing random contacts. Schools and companies are of vastly different size and the authors use available data from Oslo to inform the distribution of these entities. However, modeling large entities (schools or companies) as complete graphs where each

individual has a daily contact with each other leads to issues that may strongly influence the results.

The authors tuned their model so that the number of infections stemming from households, schools, work, etc. match Norwegian public health data (page 11 of supplement of ref 8). However, this matching means that individuals at small entities hardly contribute to the epidemic and that the epidemic is largely driven by those individuals that are part of a large clique. While this seems intuitive in general, the assumption of a complete graph and the same infection rates (Supp Table 1 in ref 8), irrespective of size of the clique, troubles me. The authors acknowledge this in page 5 of suppl of ref 8, "other layers, such as schools and workplaces, are not likely to actually see this form of all-to-all contact on any given day. This is reflected in a substantially lower combined contact and infection probability for these layers". Small schools and workplaces likely see all-to-all contacts on a given day, while large schools and workplaces do not. Since the number of contacts (edges) in a k-clique increases quadratically in k, the authors' assumption of same infection probabilities for a certain entity seems wrong and leads to a model where too much of the epidemic spread happens at large entities.

The Reviewer raises a very valid point. While the setting of different probabilities for different layers aims to account for weaker contact in larger groups, the representation of cliques as complete graphs may indeed lead to large cliques within a given layer making up an over-estimated proportion of that layer's infections. Certainly, a more detailed and accurate representation of schools and workplaces would be a great improvement to the model, and is one of the main goals for the model's ongoing development, but as things currently stand some simplifications are necessarily made. However, we do not believe that this simplification has any substantial impact on the conclusions in the manuscript. Household size is correlated with neither the size of workplaces nor that of schools. Given a working-age individual, the size of the workplace is not correlated with age. While there is some variation in size between elementary schools, middle schools and high schools, these levels are also represented as different layers, with no further correlation with age within each layer. It is therefore hard to see why the clique assumption, as long as one is willing to accept it for households, should result in undue bias towards any of the evaluated strategies.

The findings, as they stand, would be huge news. However, the manuscript lacks a sensitivity analysis that investigates how the differences shift when the society is in a strict lockdown vs normal operation.

In Figures 2 and 3, the authors vary R0, the basic reproductive number prior to vaccination (called unvaccinated R), but only by varying the random contact level. If this variation of R0 is to represent the different states of social distancing, school, work, etc contacts must also be varied. In particular, how do the results change if the society opens (as it happened in the United States) and proportionately fewer of the contacts happen at the household level? The authors only investigate a fixed reduction in contacts at each entity and it seems that the values used are no longer accurate: from their previous paper (ref 8, suppl, page 11), "all schools and day care facilities close, as do 50% of workplaces. Random contacts are reduced by 83%". Schools are no longer closed. I would be very interested in seeing a 2-D sensitivity analysis where the contact level at schools/workplaces as well as random contacts are varied.

The Reviewer raises an interesting point. Incidentally, our Alpha vs. Delta analysis is equivalent to the sensitivity analysis requested, as the simulation parameters differ in the daily risk in contracting the disease from sharing a clique with an infected individual (40% higher risk of transmission across cliques for Delta). Since transmission within cliques is abstracted, the model doesn't actually distinguish whether this elevated risk reflects a more potent pathogen or behavioral differences. As we also vary transmission in the random layer for each of the variants, we essentially already perform a 2D sensitivity analysis, with one dimension corresponding to activity in the random layer and the other corresponding to activity in the non-random layers. While the latter "axis" only covers two values, the difference between them (40%) is quite substantial and still does not seem to have a dramatic impact on the general observations made. While it could be tempting to attempt to try various independent combinations of infectivities in the non-random layers rather than a uniform relative increase, increasing the number of degrees of freedom would be computationally prohibitive, and any results would be difficult to present in a tidy manner.

We have added a paragraph in the manuscript to reflect this (lines 141-157), as we believe clarity on

this would be of benefit to readers.

Minor comments / concerns:

• *Why is the effective household size reduced by exactly one and not by the respective vaccine effectiveness, wouldn't that be a more appropriate measure?*

The Reviewer is absolutely correct in their observation, and we thank them for bringing this to our attention. The text has now been corrected to reflect this.

• *How much better would the following modified EHR strategies perform compared to EHR? Would the improvement (over random assignment of the last doses) only be marginally better?*

1. assign last doses (lines 152-155) to those households (one each) with the oldest unvaccinated individuals

2. assign last doses (lines 152-155) to those households (one each) with the highest average age

It would certainly be very interesting if the EHR strategy could be improved by making modifications such as those suggested by the Reviewer. We have now added evaluations of these strategies in the Supplementary Text, S4 Fig. Unfortunately, these seem to perform quite similarly to the original version, if not slightly worse for alternative 2.

• *How many times was the stochastic model run for each parameter setting? This needs to be mentioned*

We thank the Reviewer for bringing this omission to our attention. The underlying number of runs is now included with each figure and table.

Reviewer #2:

The authors here leverage an agent based model introduced in a previous publication to characterize COVID-19 evolution in Oslo and explore the outcome of different vaccine prioritization strategies. Within the different choices to judge the suitability of a given strategy, the authors quantify how the vaccination of different groups shapes the effective reproductive number of the disease. Specifically, they find that the random vaccination of the population seems more efficient for that purpose than following age-based strategies. Moreover, they propose another strategy, the effective household reduction (EHR), which focus on targeting those individuals belonging to largely populated households. Overall, this strategy leads to a higher decrease of the effective reproduction number and therefore less vaccination efforts to reach herd immunity in the population.

The results presented in the manuscript are novel, the paper is scientifically sound and correct and the claims made by the authors are supported by the information shown in the figures and tables. Therefore, I think that the paper meets the criteria for publication in PLOS One. Nonetheless, prior to an eventual publication, I have some suggestions to improve the readability of the manuscript and to better contextualize the findings made by the authors based on the literature recently published addressing the same problem.

We thank the Reviewer for their interest, effort and positive assessment of our manuscript.

In this sense, I think that the debate raised by the authors about the conflict between prioritizing the disruption of the transmission chains (and therefore reducing the effective reproduction number) and other strategies targeting the most vulnerable population to reduce the death toll taken by the disease would benefit from including more references. For instance, I encourage the authors to discuss the similarities and differences of their findings with respect to the results reported in [K. Bubar et al. Science 371, 6532

(2021)] or in [J. Buckner et at. PNAS, 118 (26), E2025786118 (2021)].

We thank the Reviewer for bringing these articles to our attention. We have now expanded on our discussion (in particular, lines 347-370), and believe this strengthens the manuscript.

The authors should extend the discussion on the results obtained in the manuscript. In this sense, I think that including some statistics on the number of interactions of each age group in the network of contacts would help the reader understand the roots of the failure of age-based strategies. It would be also very convenient to explain the origin of the crossover points between the random and EHR vaccination strategies observed in some scenarios. Can it be related to the fact that, when the pathogen infectiousness is high, it is more convenient to distribute vaccine across different affected households rather than targeting those with more members?

We agree with the Reviewer that including statistics on contact data is of great relevance to the manuscript. In order to keep the main manuscript concise and focused, we have included these data and associated discussion in a supplementary file to our manuscript, in S5 and S6 Fig, in addition to a discussion (lines 325-339) as to how these relate to social contact matrices in the cited papers.

I think that the distinction between Alpha and Delta variants is a bit misleading because the intervals assumed for the basic reproduction number of both variants overlap each other. Given the fact that there is no clear agreement on the specific values associated with each variant, I would avoid making this classification and just introduce the analysis of the results in general terms, addressing how the outcome of the vaccination varies as a function of the basic reproduction number.

The rationale for presenting two variants, each with a wide span of basic reproduction numbers, is that due to the lack of a period of "ordinary" behavior during the COVID-19 pandemic, the exact amount of random activity that corresponds to a full re-opening of society is uncertain. We therefore opted to try a span of increases in the activity of the random layer, in order to cover most eventualities. R_0 is not an input parameter for the model, rather it is a consequence of the level of activity set in the random layer. For a given value of R_0, the Alpha and Delta are not interchangeable, as the amount of re-opening in the Delta case will actually be lower, while the spread in the non-random layers will represent a larger share of the total.

The authors should provide more details concerning the number of realizations carried out to obtain the different results shown throughout the manuscript. Likewise, the results presented in the different tables should include the confidence intervals extracted from the simulations to improve the statistical analysis.

We thank the Reviewer for bringing this omission to our attention. We now specify the number of realizations for each table and figure, and have added confidence intervals to the data in the tables.

As a minor detail, I think that there is a typo in Table 2 regarding the herd immunity value estimated for a randomly distributed vaccine with 90% efficacy and $R_0 = 3.1$

We thank the Reviewer for spotting the typo. It has now been rectified.
