# Supplementary material for: Comparing the impact of vaccination strategies on the spread of COVID-19, including a novel household-targeted vaccination strategy
Source: PLoS One. 2022 Feb 2;17(2):e0263155. doi: 10.1371/journal.pone.0263155 (PMC8809548; doi:10.1371/journal.pone.0263155)
Supplement: Attachment — Submitted filename: Response to Reviewers.pdf [file pone.0263155.s010.pdf]

Response to Reviewers

General Comments

We thank the Reviewers for their help in improving our work, and the Editor for the chance to resubmit a further revised version to PLoS One.

Review Comments to the Author

Reviewer #1:

I want to thank the authors for adding missing details regarding the methodology. As a result, the manuscript is much improved and is in my opinion, with a few minor changes, suitable for publication in PLOS One.

Minor changes:
1. I appreciate the added explanatory supplementary figures. It would be helpful if the authors could add full figure legends (not just a caption) for each one of these figures, in particular the more complicated ones such as SFig 6.

We thank the Reviewer for bringing this to our attention. We have now added full figure legends for each supporting figure. As a minor related edit, we have added ticks to the top x-axis of S3 Fig, as we believe this makes the accompanying legend easier to contextualize.

2. The additional description of the random contact network (lines 62-71) helps but is still incomplete. It should always be the goal that, at least in theory, one can reproduce the results. This is only possible if the authors mention somewhere the used parameter values, e.g. for the normal and for the power-law distribution, and also what proportion of contacts are considered random contacts.

We have now included parameter values in our description of the random layer (lines 64-71). The proportion of infections that take place in the random is not a fixed amount, as the infectivity of the random layer is the parameter which is varied to obtain different values of R. We have expanded on the description of the handling of robustness to better reflect this (lines 151-157, 161-163).

Other comments:
1. I appreciate the additional text (line 325-339). It helps to understand why the authors chose their approach. However, I still believe this study could benefit from inclusion of POLYMOD data, at least for the random contact level, which should in theory not be hard. One example where the author's approach leads to clearly non-realistic contact patterns is the amount of contact between 14-18 and 20-24 year olds. SFig 6 shows that there are virtually no contacts between these two close age groups. This must be an artifact of the social clique approach.

We agree with the Reviewer that adding an age-based component to the random layer would be an improvement to the model, but this turns out to be computationally prohibitive with the model in its current form. We have, however, expanded on this limitation in the discussion section, which should help the reader contextualize the findings (lines 344-350).
